# Comparison of different methods for preparation and characterization of total RNA from cartilage samples to uncover osteoarthritis *in vivo*

**DOI:** 10.1186/1756-0500-3-7

**Published:** 2010-01-18

**Authors:** Anke Ruettger, Steffi Neumann, Bernd Wiederanders, René Huber

**Affiliations:** 1 Institute of Molecular Pathogenesis (IMP), Friedrich-Loeffler-Institute, Federal Research Institute for Animal Health, Jena, Germany; 2 Research Unit at the Waldkrankenhaus "Rudolf Elle", Department of Orthopaedics, University Hospital Jena, Eisenberg, Germany; 3 Institute of Diagnostic and Interventional Radiology, University Hospital Jena, Jena, Germany; 4 Institute of Biochemistry I, University Hospital Jena, Jena, Germany; 5 Institute of Clinical Chemistry, Hannover Medical School, Hannover, Germany

## Abstract

**Background:**

The isolation of intact RNA can be very difficult when tissues are used that contain many RNAses or that are hard to homogenize, e.g. cartilage samples. Additionally, cartilaginous tissues are characterized by a low cellularity and an abundance of extracellular matrix (ECM) molecules. But given the growing interest in understanding pathogenesis of degenerative diseases, e.g. osteoarthritis (OA) and rheumatoid arthritis (RA), studies have to consider expression pattern of cells in its natural environment.

**Findings:**

We compared the current RNA isolation methods for the extraction of high-quality RNA of snap-frozen biopsies from limited amounts of hypocellular cartilaginous tissue. The focus of the study was to gather information about procedure-related differences in RNA quality and yield. Here, we describe two protocols, the phenol/chloroform-free filter-based method (RN*Aqueous*™ kit) and the combined protocol (TRIzol^®^/RNeasy Mini™ kit), working in a reproducible and reliable manner.

**Conclusions:**

We conclude that preparation, storage, homogenization, and quality control are altogether critical steps for in-depth analysis of differential gene expression, especially in hypocellular tissues with highly crosslinked ECM like cartilage.

## Findings

### Context

Experimental investigation of chondrocytes in their physiological environment is a matter of particular interest in the field of rheumatic diseases, such as osteoarthritis (OA) and rheumatoid arthritis (RA). The isolation of sufficient amounts of high quality RNA from articular cartilage is a persistent problem for the research addressing cartilage molecular biology [1-5]. This is particularly caused by three factors: *(i) *cartilage shows a low cell content (only 1 - 5% of total mass), *(ii) *it is characterized by a highly crosslinked extracellular matrix (ECM) containing large amounts of proteoglycans, and *(iii) *considerably enhanced levels of RNA degradation in human cartilage samples obtained from arthritis patients.

**Figure 1:**
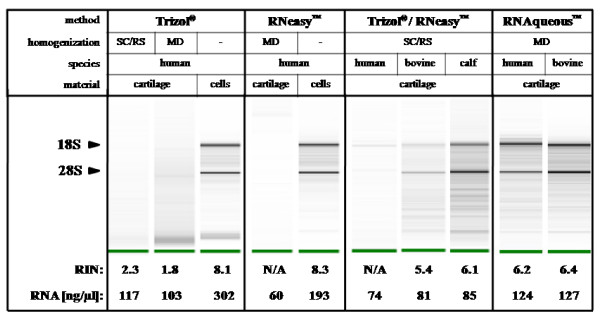
**Comparison of current methods for total RNA isolation from the small cell tissue cartilage: quality control using capillary electrophoresis**. Total RNA was isolated from chondrocytes (cells) and cartilage explants. Cartilage homogenization was performed with scalpel (SC), rotor-stator (RS) or microdismembrator (MD). Different extraction procedures (TRIzol^®^, RNeasy™, TRIzol^®^/RNeasy™ and RNA*queous*™) were performed as described in the Methods section. Integrity of RNA isolated from different species (human, adult bovine cartilage - cow, and immature bovine cartilage - calf) was analyzed by capillary electrophoresis. 18S and 28S rRNA bands correspond to 41-43 and 47-50 [s], respectively. The RNA yield is specified for each sample [in ng/μl]. After precipitation and washing the RNA was resuspended in different volumes of RNase-free water: 30 μl (Trizol^®^), 20 μl (RNeasy™), 10 - 20 μl (Trizol^®^/RNeasy™) and 10 - 20 μl (RNA*queous*™). On account of this, the results provided here are comparable with the results provided in Table 1 (μg RNA per 100 mg cartilage). The results are shown by gel electrophoresis (RIN: RNA integrity number; N/A: not available).

Our objective was to develop a detailed code of practice for successful high quality RNA isolation from cartilage samples. While it is generally accepted that RNA purification from chondrocytes is quite simple resulting in large amounts of high quality RNA [Figure 1, Additional file 1], there is no "gold standard" for the isolation of total RNA from cartilage samples [6,7]. Although several protocols are available for RNA isolation from cartilage [4,8,9], these studies did not show adequate proofs of RNA quality. Recently, Geyer et al. [2] described a phenol/chloroform-free filter-based system for successful RNA isolation from cartilage disrupted in a guanidinium thiocyanate solution (RNA*queous*™). In contrast to former studies, a solid proof of RNA quality using capillary electrophoresis was included. Therefore, our main objective was to compare this new approach with standard protocols using TRIzol^® ^reagent and/or the RNeasy™ system with respect to procedure- and species-related differences in RNA quality and yield.

## Materials and methods

Human cartilage samples were obtained from patients undergoing total knee replacement surgery. Tissue samples were used with patients' informed consent and institutional approval (Friedrich Schiller University Jena, Medical Faculty Ethics Committee approval 1772-04/06). Adult bovine (cow) and immature bovine (calf) cartilage was directly obtained from the slaughterhouse. Sterile cuts (diameter 3 mm, e.g., 50 mg tissue per tube) were prepared with a cutter (B. Braun/Aesculap, Melsungen, Germany) from cartilage samples. Immediately after removal, the cartilage cuts were frozen in liquid nitrogen and thereafter stored at -80°C (for long-time storage).

Detailed protocols for RNA isolation with the RNAqueous™ kit and with TRIzol^®^/RNeasy Mini™ kit are provided in the Additional files [see Additional files 2 and 3]. Moreover, reduction of the RNA solution volume with GlycoBlue™ Coprecipitant is described [see Additional file 2].

For detection of RNA quality, RNA yield, A_260_:A_280_, and A_260_:A_230 _ratios were determined using the NanoDrop^® ^ND-1000 spectrophotometer (NanoDrop Technologies, Wilmington, DE, USA). RNA integrity was determined by measuring 28S/18S rRNA ratios and calculating the respective RNA integrity number (RIN) on an Agilent 2100 Bioanalyzer using the RNA 6000 Nano LabChips (Agilent Technologies, Santa Clara/Palo Alto, CA, USA). Synthesis of cDNA and (q)RT-PCR were performed as previously described [[7,10], see Additional file 4].

Statistical analyses were performed using the statistical software SPSS 10.0 for Windows (SPSS Inc., Chicago, IL, USA). Data were analyzed using the Mann-Whitney *U *test. Values of *p *≤ 0.05 (*) were considered statistically significant.

## Key points

Researchers dealing with the "special" tissue cartilage have to consider several guidelines and pitfalls for an isolation of high quality RNA and expedient downstream applications:

*(i) *Adequate transport and storage conditions for cartilage samples. An immediate sample transfer, transport, and storage in liquid nitrogen are normally well-established. On the other hand, storage of cartilage in a stabilization reagent like RNAlater™ is not advisable. Such reagents cause dehydration of the cartilage resulting in relevant problems during subsequent homogenization using a microdismembrator: the cartilage becomes extremely rigid. Only if a scalpel is used for dissection, RNAlater^® ^does not negatively influence the homogenization process, but offers no benefit compared to storage of the cartilage explants at -80°C [see Additional file 5].

*(ii) *Appropriate cartilage homogenization. Our study also links the isolation of high quality RNA to an appropriate cartilage homogenization method [see Additional files 1 and 5]. The available tissue homogenization methods can not necessarily be combined successfully with any existing RNA isolation protocols [see Figure 1 and Additional file 1].

*(iii) *Application of the RNA*queous*™-based method for human cartilage samples. Our study shows that at the moment, this method provides the best results for the RNA isolation from human cartilage samples [see Table 1].

**Table 1:** Variation of Ct-values of specific cartilage genes due to using different RNA isolation methods.

	Ct-values (mRNA-Expression)				
			
METHOD	Col II (109 bp)	Col II (609 bp)	GAPDH (254 bp)	**ΔCt**_1_	**ΔCt**_2_
Trizol^®^	23.1	28.9	23.5	-0.4	5.4
RNeasy™	23.2	28.5	23,7	-0.5	4.8
Trizol^®^/RNeasy™	23.2	26.8	23.8	-0.6	3.0
RNA*queous*™	24.1	24.5	23.5	0.6	1.0

One human cartilage sample was obtained from an OA patient (69 years old) undergoing total knee replacement surgery. Sterile cuts (4 × 50 mg tissue) were prepared and stored in liquid nitrogen. The cartilage samples were reduced to powder using a microdismembrator. After that RNA was isolated according to the respective protocol. After isolation, 0.2 μg RNA was reversed transcribed into cDNA. 1 μl of the cDNA was used as template to amplify human collagen type II messages. QRT-PCR was carried out using a MyiQ Cycler with the iQ SYBR Green Supermix, as described by the manufacturer (Bio-Rad).

*(iv) *Mandatory check of RNA integrity by using capillary electrophoresis. In general, the RIN value should be ≥ 6.0 for downstream applications, such as cDNA synthesis (or even ≥ 8.0 for chip analysis and qRT-PCR). Empirically, using the established isolation protocols, RIN values ≥ 7.0 could not be achieved. However, our experiments [see Figure 1] show that the implementation of the protocols described above yields in RNA qualities sufficient for sensitive downstream applications, e.g. qRT-PCR.

*(v) *Intelligent primer design. This section has to be considered to exclude partial RNA degradation and/or DNA impurities. The results shown in Table 1 also underline in this requirement.

*(vi) *Careful interpretation of studies dealing with the topic gene expression from cartilage. Since a variety of studies is based on suboptimal RNA extraction methods, the results of these studies have to be handled with care [see Table 1].

*(vii) *Consideration of species-related and quality-related differences among cartilage samples. Our results clearly implicate species-related problems with RNA isolation from cartilage. Most of the human cartilage samples obtained from patients were reduced in thickness and showed defects of the cartilage layer due to pre-existing disease [data not shown]. In contrast, cartilage samples derived from cows or calfs are characterized by an enhanced cell number. For these samples, even other extraction methods are suitable. In addition, the RNA extraction from cartilage samples of healthy donors appears much easier in comparison to material obtained from diseased donors.

## Discussion and future development

Concerning an adequate storage condition for maintaining RNA integrity recent studies suggest the incubation of freshly obtained cartilage samples in the stabilization reagent RNAlater™ [11-13]. Here, we have to emphasise that the use of RNAlater™ results in a considerable curing process, thus impeding homogenization with a ball mill.

Concerning the method of choice for cartilage homogenization, we have to conclude that the respective method should be chosen in dependency of the subsequent isolation method. For example, milling before RNA isolation with TRIzol^® ^reagent and RNeasy™ resulted in a significant RNA degradation [see Figure 1 and Additional files 1 and 5]. Consequently, gene expression profiling based on the RNA isolation with TRIzol reagent following milling dramatically yields in false positive results [see Table 1 and Additional file 6]. Interestingly, subsequent application of the RNA*queous*™ system, however, was not negatively influenced by milling regarding both RNA yield and quality [see Figure 1 and Additional file 1]. Indeed, in comparison with the other three methods addressed in our study, the application of the RNA*queous*™ kit system further increases the quality and especially the amount of isolated RNA in any circumstance [see Figure 1, Tables 1 and 2 and Additional file 1]. These results show that the RNA isolation method determines the appropriate method for cartilage homogenization.

**Table 2:** Recommendation for the use of different methods for RNA isolation from cartilage/chondrocytes.

METHOD	MATERIAL			
	**chondrocytes**	**comments**	**cartilage**	**comments**
	
Trizol^®^	**++**	solid RNA quality	**-**	RNA degradation
		high RNA yield		
RNeasy™	**+++**	high RNA quality	**-**	RNA degradation
		solid RNA yield		
Trizol^®^/RNeasy™	**+**	cost-benefit ratio (-)	**+/++**	solid RNA quality
		solid RNA quality		*(difficulties with human samples)*
		solid RNA yield		solid RNA yield
RNA*queous*™	**+**	cost-benefit ratio (-)	**+++**	high RNA quality
		solid RNA quality		solid RNA yield
		solid RNA yield		

The results/criteria for recommendation are given as follows: - fail; + passing; ++ good; +++ very good.

A matter of special importance is the mandatory check of RNA integrity of each sample by using capillary electrophoresis [see Figure 1 and Additional file 5]. The RIN classifies the integrity of eukaryotic total RNA on a scale of 1 to 10 (most to least degraded) based on the electropherogram of the 18S and 28S rRNA peaks, thus adequately indicating evidence for potential RNA degradation.

In order to exclude that RNA degradation in human material was due to the pathologies of the patients from whom the samples originated, we also examined "healthy" cartilage derived from young (calves) and old cows. We found species-related and quality-related differences [see Additional file 1]. Indeed, the number of chondrocytes per gram tissue is significantly reduced in human compared to bovine cartilage [see Additional file 1]. The cell-rich cartilage of calves showed the highest yield of non-degraded RNA, whereas the cartilage of adult cows and, moreover, the cartilage samples of diseased patients showed severe signs of degradation [see Figure 1].

As expected, RNA isolated from cartilage does never reach yield and purity of RNA isolated from cultured chondrocytes [see Additional file 1]. However, the new RN*Aqueous™*-based method should be useful for identifying genes differentially expressed by chondrocytes in its natural environment. In spite of this advance, there is still room for improvements and recent studies have to be interpreted with care.

## Abbreviations

ECM: extracellular matrix; gDNA: genomic DNA; MD: microdismembrator (ball mill); OA: osteoarthritis; PCR: polymerase chain reaction; QC: quality control; RT-PCR: quantitative Real-time-PCR; RA: rheumatoid arthritis; RIN: RNA integrity number; RS: rotor-stator; RT-PCR: reverse transcription polymerase chain reaction; RT: room temperature; SC: scalpel.

## Competing interests

The authors declare that they have no competing interests.

## Authors' contributions

AR designed the study. AR and SN planned and conducted the laboratory experiments. AR, RH and BW analyzed the data. The manuscript was drafted by AR and edited by AR and RH. All authors approved the final manuscript.

## Supplementary Material

Additional file 1 **Comparative analysis of current methods for RNA isolation from cartilage/chondrocytes**. This table provides RNA quality control parameters (detected with the NanoDrop) and parameters of cell yields after chondrocyte extraction from cartilage. Click here for file

Additional file 2 **Protocol 1 - RNA isolation from cartilage using RNA*queous *Midi™ kit**. This data file provides a complete protocol for using the RNA*queous *Midi™ kit. It enables the reader to start immediately with RNA isolation. This protocol is the best one for RNA isolation from human cartilage samples. Click here for file

Additional file 3 **Protocol 2 - Combined method for RNA isolation from cartilage**. This data file provides a complete protocol for using the combined method. It enables the reader to start immediately with RNA isolation. This protocol is acceptable for RNA isolation from bovine cartilage samples. Click here for file

Additional file 4 **Primers, product length, and specific amplification conditions for (q)RT-PCR**. This table provides additional information about primers and amplification conditions for qRT-PCR and RT-PCR. Click here for file

Additional file 5 **Characterization of special parameters during RNA isolation from bovine articular cartilage**. In this figure we compare special parameters during RNA isolation based on Agilent analysis. Click here for file

Additional file 6 **Quality control of total RNA from human cartilage explants from one typical donor using RT-PCR**. In this figure, we present the results of an typical gel electrophoresis image after RT-PCR. Click here for file
