# Supplementary material for: Comparison of different methods for preparation and characterization of total RNA from cartilage samples to uncover osteoarthritis in vivo
Source: BMC Res Notes. 2010 Jan 18;3:7. doi: 10.1186/1756-0500-3-7 (PMC2841606; doi:10.1186/1756-0500-3-7)
Supplement: Additional file 1 — Comparative analysis of current methods for RNA isolation from cartilage/chondrocytes. This table provides RNA quality control parameters (detected with the NanoDrop) and parameters of cell yields after chondrocyte extraction from cartilage. [file 1756-0500-3-7-S1.PDF]

Additional file 1. **Comparative analysis of current methods for RNA isolation from cartilage/chondrocytes**

a. RNA quality control parameters – NanoDrop data

			CARTILAGE			CHONDROCYTES		
Method	***Homogenization***	***n***	A_{260}:A_{280}	A_{260}:A_{230}	***RNA yield***	A_{260}:A_{280}	A_{260}:A_{230}	***RNA yield***
Trizol®	SC / RS	10	**1.75** (0.89 – 3.47)	**0.26**	**1.32** (0.56-3.61)	**1.91** (1.19 – 2.37)	**1.20**	**12.74** (3.32 – 37.16)
	MD	10	**1.49** (1.06 – 1.76)	**0.16**	**3.21***(2.98-3.54)			
RNeasy™	MD	10	**1.36** (0.35 – 3.65)	**0.11**	**0.49** (0.24-1.23)	**2.01*** (1.93 – 2.04)	**1.42**	**3.44** (0.48 – 9.43)
Trizol®/RNeasy™	SC / RS	10	**2.12** (1.48 – 3.36)<br>**1.79*** (1.62 – 1.98) #	**0.93**<br>**0.59** #	**0.23** (0.14-0.80)<br>**0.92** (0.52-1.14)#			
RNA*queous*™	MD	10	**1.78*** (1.68 – 1.85)<br>**1.80*** (1.72 – 1.93) #	**0.86**<br>**1.00** #	**0.64** (0.44-1.24)<br>**0.80** (0.42-1.33)#			

b. Parameter cell yields after chondrocyte extraction from cartilage

Material	***Species***	***n***	***Cell yield [10^6 chondrocytes per g cartilage]***
cartilage	human	20	4.75 (2.38 – 7.06)
cartilage	bovine	10	20.68 (10.7 – 21.57) #

Comparative analysis of different RNA isolation methods was performed using Trizol®, RNeasy™ Mini, Trizol®/RNeasy™ Mini and RNA*queous-Midi*™. Different homogenization variants were performed (scalpel_SC; microdismembrator_MD; rotor-stator_RS). The results are given as average values (*bold*) and the range of values *(in brackets)*. RNA yield is specified in µg per 100 mg cartilage. After precipitation and washing the RNA was resuspend in different volumes of RNAse-free water: 30µl (Trizol®), 20 µl (RNeasy™), 10 - 20 µl (Trizol®/RNeasy™) and 10 – 20 µl (RNA*queous*™*)*. On account of this, the results provided here are comparable with the results provided in Figure 1 (ng RNA per µl). The cell yield is specified in million chondrocytes per g cartilage. In general, the results are given as average values (*bold*) and the range of values *(in brackets)*. Results of RNA isolation and cell extraction from bovine cartilage were marked with #. Analyses were performed with statistical software SPSS 10.0 for Windows (SPSS Inc., Chicago, IL, USA). Data were analyzed with Mann-Whitney *U*-test. Values of * $p<0.05$ were considered significant.
