# Supplementary material for: Comparison of different methods for preparation and characterization of total RNA from cartilage samples to uncover osteoarthritis in vivo
Source: BMC Res Notes. 2010 Jan 18;3:7. doi: 10.1186/1756-0500-3-7 (PMC2841606; doi:10.1186/1756-0500-3-7)
Supplement: Additional file 2 — Protocol 1 - RNA isolation from cartilage using RNAqueous Midi™ kit. This data file provides a complete protocol for using the RNAqueous Midi™ kit. It enables the reader to start immediately with RNA isolation. This protocol is the best one for RNA isolation from human cartilage samples. [file 1756-0500-3-7-S2.PDF]

Detailed protocol for RNA isolation from cartilage using RNAqueous Midi™ kit

REAGENTS

- RNAqueous Midi™ kit (AM1911):
    - kit components: glass fiber filter units, lysis/binding solution, nuclease-free water in container suitable for preparing 64% ethanol solution, wash solution #1, wash solution #2/3 concentrate, elution solution, RNase-free collection tubes
- 100 % Ethanol (ACS grade)
- GlycoBlue™ Coprecipitant (AM9515 or AM9516, Ambion, Inc., Austin, Texas, USA)
- Ammonium acetate (Roth, Germany)
- RNA Nano kit 6000 (Agilent Technologies, Santa Clara/Palo Alto, CA, USA)
- RNase-free water (see recipe)

PROCEDURE

We recommend to wear gloves and to use RNase-free tips and tubes for all procedure steps!

PREPARATION AND STORAGE

1. Prepare sterile cuts from human cartilage explants (diameter 3 mm, e.g., 50 mg tissue per tube). Use a scalpel and a cutter for cartilage preparation.

2. Store the cartilage cuts immediately in liquid nitrogen and then at -80°C until RNA extraction.

ATTENTION:

It is not advisable to store cartilage explants in RNAlater™, because the cartilage becomes very hard.

HOMOGENZATION

MICRODISMEMBRATOR (MD)

1. Store the vial in liquid nitrogen. Pre-cool the ball mill impactor also in liquid nitrogen.
2. Place the cartilage in the ball mill and store immediately for at least 5 minutes in a liquid nitrogen-cooled box .

3. Reduce the explants to a powder in a microdismembrator (B. Braun Micro-Dismembrator, 1 minute, shaking rate: 2000 rpm).
4. Place the powder uninterrupted in at least 12 volumes Lysis Buffer (we resuspend 100 mg cartilage in 1.3 ml lysis buffer) and store the sample at least over night at -80°C .

RNA ISOLATION

Necessary preparations (A-C):

A. Prepare *once* 64% Ethanol (add 64ml 100% ethanol to the bottle containing 36ml RNase-free water.

B. Prepare *once* wash solution #2/3 (add 201.6ml 100% ethanol to the wash solution #2/3 concentrate).

C. Before starting heat elution solution *always* to ~100°C in a heat block (e.g., 0.6 ml per sample).

RNA-Isolation:

1. Incubate homogenized cartilage samples for 30 minutes at room temperature. Centrifuge for 5 minutes (4°C, 5 000 g). Transfer the supernatant into a RNase-free tube.
2. Add an equal volume of 64% ethanol and shake the tube vigorously by hand (at least 3 times).
3. Pass the lysate mixture through a glass fiber filter as follows:
    a. Attach an 18 gauge needle to a large disposable syringe (5ml or 10 ml).
    b. Aspirate the the lysate up into the syringe through the needle.
    c. Remove the needle from the syringe, attach a glass fiber unit and slowly pass the lysate through the filter (~ 3-5 drops per second).
4. Wash the glass fiber filter unit with the Wash solution #1.
5. Wash the glass fiber filter unit twice with the Wash solution #2,3.
6. Important step: Remove the filter unit, retract the plunger of the syringe, and vigorously force air through the filter to remove residual wash solution. Repeat this step until no additional droplets or fine spray is visible (~5 X).
7. Pass 300 µl of hot elution solution through the filter. Repeat this step once again.

Standard salt/ethanol nucleic acid precipitation with GlycoBlue™ Coprecipitant:

a. Prepare a 50 M ammonium acetate solution (3.8 g per ml).
b. Add ammonium acetate to 0.5 M (1:100 dilution).

c. Add GlycoBlue™ Coprecipitant to 50 µg/ml.

d. Add two volumes 100% ethanol.

Example for preparing the ClycoBlue™ Coprecipitation solution:

~600 µl eluat (2 x ~300 µl)

+ 20 µl 50M ammonium acetate

+ 1 µl GlycoBlue™

+ 1300 µl 100% Ethanol

e. Incubate at -20°C for 15 min and centrifuge at top speed (~13 000 rpm) in a microcentrifuge for 15 min at room temperature. (Note: It is possible to store the sample at -20° C for a longer period, e.g., 3 days.)

f. Heat RNase-free water to 65°C and

g. Discard the supernatant and resuspend the blue pellet in about 10 to 20 µl RNase-free water.

RNA QUALITY CONTROL METHODS

AGILENT 2100 BIOANALYZER

1. Analyze RNA integrity for example with RNA 6000 Nano LabChips. Perform analyze according to manufacturers' instructions (for detailed information: *www.chem.agilent.com*).

TROUBLESHOOTING - Detailed information is available online (*www.ambion.com*).

EQUIPMENT

- Large and small calibrated disposable syringes (3ml and 10ml)
- 18 gauge 1.5 inch needles
- Agilent bioanalyzer 2100 (Agilent Technologies, Santa Clara/Palo Alto, CA, USA)
- cutter (cat. no. OL670R, Aesculap/B. Braun, Melsungen, Germany)
- Microdismembrator (B. Braun, Melsungen, Germany)
- RNase-free tubes and tips
