# Supplementary material for: Comparison of different methods for preparation and characterization of total RNA from cartilage samples to uncover osteoarthritis in vivo
Source: BMC Res Notes. 2010 Jan 18;3:7. doi: 10.1186/1756-0500-3-7 (PMC2841606; doi:10.1186/1756-0500-3-7)
Supplement: Additional file 3 — Protocol 2 - Combined method for RNA isolation from cartilage. This data file provides a complete protocol for using the combined method. It enables the reader to start immediately with RNA isolation. This protocol is acceptable for RNA isolation from bovine cartilage samples. [file 1756-0500-3-7-S3.PDF]

Detailed protocol: Combined method for RNA isolation from cartilage

REAGENTS

- chloroform
- DNase (RNase-free DNase Set, cat.no. 79254, Qiagen, Hilden, Germany)
- 80 % Ethanol (in DEPC-treated water)
- ethidium bromide (EtBr)
- H_2O_2
- RNAlater™ (cat. no. 76104, Qiagen, Hilden, Germany)
- TRIzol® reagent (cat. no. 15596-018, Invitrogen, Carlsbad, CA, USA)
- RNA Nano kit 6000 (Agilent Technologies, Santa Clara/Palo Alto, CA, USA)
- RNase-free water (see recipe)
- SYBR Green II RNA stain (cat. no. S-7564, Molecular Probes, Eugene, OR, USA)

PROCEDURE

We recommend to wear gloves and to use RNase-free tips and tubes for all procedure steps!

PREPARATION AND STORAGE

1. Prepare sterile cuts from human cartilage explants (diameter 3 mm, e.g., 50 mg tissue per tube). Use a scalpel and a cutter for cartilage preparation.

2. Store the cartilage cuts immediately in liquid nitrogen and then at -80°C until RNA extraction.

ATTENTION:

A) It is not advisable to store the cuts in TRIzol® (TRIzol® does not reach the chondrocytes).

B) If you prefer homogenization with a ball mill it is not advisable to store cartilage explants in RNAlater™, because the cartilage becomes very hard.

HOMOGENZATION OPTIONS

OPTION 1: SHEAR/ SCISSORS (S)

1. Add 1ml TRIzol® reagent to the vial.
2. Clean the shear by flame. Cut the cartilage explants to very small pieces and perform total RNA isolation.

(**Note**: After shear homogenization the phenol-chloroform phase is yellow and not red!)

*OPTION 2: **R**OTOR-**S**TATOR HOMOGENIZER (RS)*

1. Add 1ml TRIzol® reagent to the cartilage explants and homogenize with a rotor-stator homogenizer and OmniTip adapter for 5 x 6 seconds. Perform this step at room temperature.
2. Add 1ml TRIzol® reagent and perform total RNA isolation.

*OPTION 3: **SC**ALPEL (SC)*

3. Cut cartilage explants in very small pieces (as small as possible) with a scalpel on ice.
4. Add 1ml TRIzol® reagent and perform total RNA isolation.

*OPTION 4: **M**ICRO**D**ISMEMBRATOR (MD)*

ATTENTION: Prefer options 1 - 3 for homogenization from articular cartilage! Option 4 reduces the cartilage to a powder and supports RNA degradation.

1. Store the vial in liquid nitrogen. Pre-cool the ball mill impactor also in liquid nitrogen.
2. Place the cartilage in the ball mill and store immediately for at least 5 minutes in a liquid nitrogen-cooled box (note: the use of special cryo-vials, e.g., from Nalgene is recommended.)
3. Reduce the explants to a powder in a microdismembrator (B. Braun Micro-Dismembrator, 1 minute, shaking rate: 2000 rpm).
4. Place the powder uninterrupted in TRIzol® and perform total RNA isolation.

RNA ISOLATION

1. Incubate homogenized cartilage samples for 15 minutes at room temperature. Centrifuge for 3 minutes (4°C, 12 000 g). Transfer the supernatant into two RNase-free tubes.
2. Incubate for 5 minutes at RT and add 180 µl chloroform per 1ml TRIzol®. Shake tubes vigorously by hand for 15 seconds and incubate them for 3 minutes at room temperature. Following centrifugation (15 min, 4°C, 12 000 g), transfer the aqueous phase in a fresh tube.
3. Add 280 µl ethanol and mix gently. Load the sample (maximum volume: 700 µl) to the RNeasy™ Mini column. Centrifuge (60 seconds, 8 000 g) and repeat as occasion demands. Discard the flow-through.
4. **Optional step** DNAse treatment: *Mix 70 µl Buffer RDD and 10 µl DNase I stock (RNase-free DNase set, Qiagen). Add the DNase I incubation mix on the column and incubate for 15 minutes at room temperature. Continue with step 6.*
5. Add 500 µl Buffer RW1 to the column and centrifuge (60 seconds, 8 000 g). Discard the flow-through.
   HINT: Discard collection tube and use a new one.
6. Add 500 µl Buffer RPE to the column and centrifuge (60 seconds, 8 000 g). Discard the flow-through.
7. Add 500 µl 80% ethanol to the column and centrifuge (30 seconds, 8 000 g). Discard the flow-through.
8. Centrifuge (3 min, 12 000 g) to dry the column.
9. Add 12 µl RNase-free water and centrifuge (60 seconds, 10 000 g) to eluate the RNA. Repeat this step again.

RNA QUALITY CONTROL METHODS

AGILENT 2100 BIOANALYZER

1. Analyze RNA integrity for example with RNA 6000 Nano LabChips. Perform analyze according to manufacturers' instructions (for detailed information: *www.chem.agilent.com*).

RNA GEL ELECTROPHORESIS

ATTENTION: Performing RNA gel electrophoresis does not compensate analysis for the Agilent 2100 bioanalyzer.

1. Wash gel apparatus with mild detergent. Rinse with DEPC-water. Dilute 30% H_2O_2 to 3%. Fill apparatus with 3% H_2O_2. Submerge comb and dams. Treat for 10 minutes. Pour off all of the H_2O_2-solution. Rinse apparatus three times with DEPC-water.
   After each rinse, pour off all DEPC-water (small amounts of residual H_2O_2 will degrade RNA!).

2. Place a 5% Ready Gel TBE-Urea gel in apparatus, submerge gel with 1x TBE Running Buffer.
   Do not add EtBr or SYBR Green II to gel or running buffer.

   Rinse out slots well with Running buffer before sample loading.

3. Incubate RNA solution with 2x RNA Loading Buffer (e.g. 20 µL RNA + 10 µL Loading Buffer) at 65°C for 10 minutes. Chill on ice. Spin briefly. (**Advice:** check gel conditions with a positive control, e.g, RNA isolated from cells).
4. Run gel at 180 V until dye runs out.
5. Stain the gel with EtBr or SYBR Green II:

   EtBr staining (RNA yield > 1µg): To stain, gently agitate for 30-60 minutes in EtBr Staining Buffer. Destain gel for 20 minutes with DEPC-water to improve contrast.

   SYBR Green II staining (RNA yield < 1µg): To stain, gently agitate for 40 minutes in SYBR Green II Staining Buffer (keep in the dark).

6. Photograph the gel.

RECIPES

DEPC-treated water = RNase-free water (2 liter)

0.1% diethylpyrocarbonate

add high purity dH_2O to 2 liter

shake over night at room temperature

autoclave

TBE Running Buffer (1 liter)

89 mM Tris base

89 mM Boric acid

2 mM EDTA

pH 8.0

add DEPC-water to 1 litre

<u>2x RNA Loading Buffer (10 ml)</u>

45 mM Tris base

45 mM Boric acid

2 mM EDTA

6% Ficoll Type 400

0.005% Bromphenol blue solution

0.025% Xylene Cyanol

3.5 M Urea

Add DEPC-water to 10 ml (store at +4°C)

<u>SYBR Green II Staining Buffer (200 ml)</u>

89 mM Tris base

89 mM Boric acid

1 mM EDTA

pH 8.0 (very important)

1:5000 SYBR Green II RNA Stain Solution

add DEPC-water to 200 ml

<u>EtBr Staining Buffer (200 ml):</u>

0.01 M NH_4OAc

1-5 µg / ml EtBr

DEPC-water to 200 ml

TROUBLESHOOTING

Detailed information is available online (for TRIzol® reagent: *www.invitrogen.com* and for RNeasy™ Mini kit *www.qiagen.com*).

EQUIPMENT

- Agilent bioanalyzer 2100 (Agilent Technologies, Santa Clara/Palo Alto, CA, USA)
- cutter (cat. no. OL670R, Aesculap/B. Braun, Melsungen, Germany)
- Gel imager (e.g., Fluor-S MultiImager, Bio-Rad, Philadelphia, PA, USA)
- Microdismembrator (B. Braun, Melsungen, Germany)
- Mini-PROTEAN II (cat. no. 161-1501, Bio-Rad, Philadelphia, PA, USA)
- Ready Gel TBE-Urea Gel (cat. no. 161-1115, Bio-Rad, Philadelphia, PA, USA)
- RNase-free tubes and tips
- Rotor-stator homogenizer & OmniTip adapter (OMNI TH International, Sued-Laborbedarf, Munich, Germany)
- Scalpel (cat.no. BA211, Aesculap, Tuttlingen, Germany)
- Sterile SYSTEM 100™ Cryogenic vials (cat.no. 5000-1012, Nalgene® Labware, Rochester, NY, USA)
