# Supplementary material for: Comparison of different methods for preparation and characterization of total RNA from cartilage samples to uncover osteoarthritis in vivo
Source: BMC Res Notes. 2010 Jan 18;3:7. doi: 10.1186/1756-0500-3-7 (PMC2841606; doi:10.1186/1756-0500-3-7)
Supplement: Additional file 4 — Primers, product length, and specific amplification conditions for (q)RT-PCR. This table provides additional information about primers and amplification conditions for qRT-PCR and RT-PCR. [file 1756-0500-3-7-S4.PDF]

Additional file 4. **Primers, product length, and specific amplification conditions for (q)RT-PCR.**

Gene	Primer Sequence (5' → 3')	PCR product (mRNA)	PCR product (gDNA)	Annealing (°C)	Species
hCol2A1	CAACACTGCCAACGTCCAGAT<br>CTGCTTCGTCCAGATAGGCAAT	107 bp	107 bp	60	human
Aggr*	ACTTCCGCTGGTCAGATGGA<br>TCTCGTGCCAGATCATCACC	111 bp	906 bp	55	human<br>bovine
GAPDH	CATCACTGCCACCCAGAAGA<br>CCTGCTTCACCACCTTCTTG	254 bp	254 bp	60	human<br>bovine
Col2A1*	CATCTGGTTTGGAGAAACCATC<br>GCCCAGTTCAGGTCTCTTAG	606 bp<br>600 bp	1482 bp	60	human<br>bovine

General amplification protocol for RT-PCR (35 cycles): initial denaturation for 3 min at 95°C; denaturation for 30s at 94°C; specific primer annealing temperature (see table) for 45s, amplification at 72°C for 45 s. An intron-spanning primer pair is marked with *. QRT-PCR was performed as described in the methods section.
