# Supplementary material for: Comparison of different methods for preparation and characterization of total RNA from cartilage samples to uncover osteoarthritis in vivo
Source: BMC Res Notes. 2010 Jan 18;3:7. doi: 10.1186/1756-0500-3-7 (PMC2841606; doi:10.1186/1756-0500-3-7)
Supplement: Additional file 5 — Characterization of special parameters during RNA isolation from bovine articular cartilage. In this figure we compare special parameters during RNA isolation based on Agilent analysis. [file 1756-0500-3-7-S5.PDF]

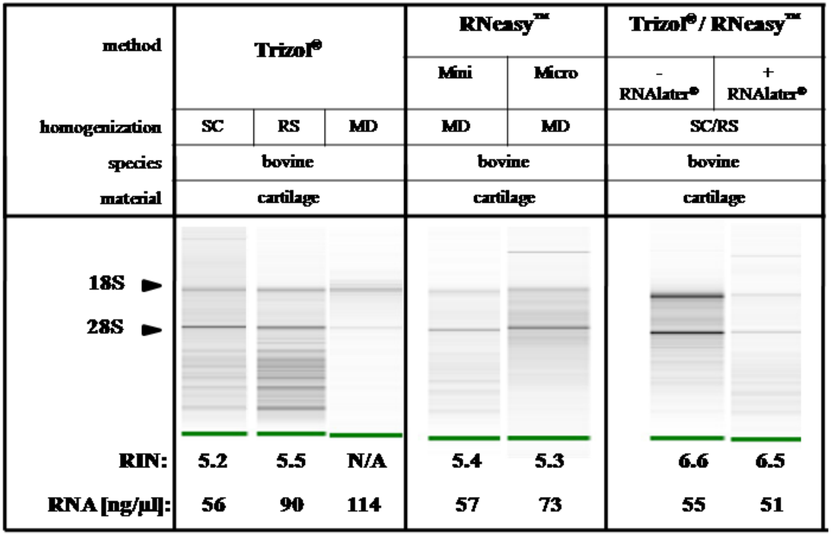

Additional file 5. **Characterization of special parameters during RNA isolation from bovine articular cartilage.**

Total RNA was isolated from bovine cartilage explants. 18S and 28S rRNA bands correspond to 41–43 and 47–50 [s], respectively. The exact RNA yield for each sample [in ng/µl] is given. Each well contained 1µl of extracted RNA from equal amount of starting materials of 100 mg cartilage. Following parameters were checked by capillary electrophoresis: left panel: analysis of the influence of different homogenization variants (rotor-stator: RS; scalpel: SC; micodismembrator: MD) on RNA integrity; middle panel: comparison of RNeasy™ Mini and Micro kits; right panel: no benefit of cartilage storage in RNAlater™. (RIN: RNA integrity number; N/A: not available).
