# Supplementary material for: Comparison of different methods for preparation and characterization of total RNA from cartilage samples to uncover osteoarthritis in vivo
Source: BMC Res Notes. 2010 Jan 18;3:7. doi: 10.1186/1756-0500-3-7 (PMC2841606; doi:10.1186/1756-0500-3-7)
Supplement: Additional file 6 — Quality control of total RNA from human cartilage explants from one typical donor using RT-PCR. In this figure, we present the results of an typical gel electrophoresis image after RT-PCR. [file 1756-0500-3-7-S6.PDF]

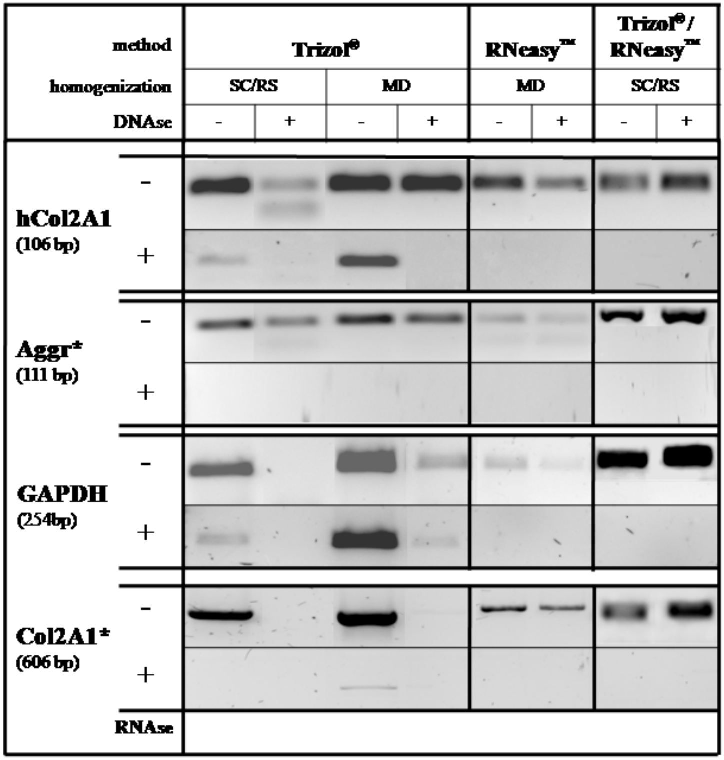

Additional file 6. **Quality control of total RNA from human cartilage explants from one typical donor using RT-PCR.** Total RNA was isolated using different homogenization options (SC: scalpel; RS: rotor-stator; MD: microdismembrator) and different extraction procedures (TRIzol® reagent and / or RNeasy Mini™ kit; with or without DNAse-treatment). The quality of human cartilage RNA was assessed by rt-PCR amplification of different genes. Total RNA was reverse transcribed into cDNA and amplification of human collagen Type II (hCol2A1; short PCR product size), aggrecan (Aggr; short PCR product, intron-spanning primer), the housekeeping gene glyceralaldehyde 3-phosphate dehydrogenase (GAPDH, middle PCR product) and collagen Type II (Col2A1, intron-spanning primer, long PCR product) was performed. As controls, RNA was pre-treated with RNase A before the first-strand synthesis, and the reverse transcriptase was omitted. PCR products were visualized by gel electrophoresis.
